# Dynamic relationships between bilirubin concentrations and the gut microbiota in the neonatal period: A pilot prospective cohort study

**DOI:** 10.1002/ped4.70032

**Published:** 2025-12-05

**Authors:** Zhongyuan Li, Yan Zhang, Xi Luo, Yangyang Wang, Lihua Peng, Liping Zou

**Affiliations:** ^1^ Medical School of Chinese PLA Beijing China; ^2^ Senior Department of Pediatrics, the Seventh Medical Center Chinese PLA General Hospital Beijing China; ^3^ Department of Gastroenterology and Hepatology, The First Medical Center Chinese PLA General Hospital Beijing China; ^4^ Department of Pediatrics, The First Medical Center Chinese PLA General Hospital Beijing China; ^5^ Department of Pediatrics, Beijing Institute for Brain Disorders, Center for Brain Disorders Research Capital Medical University Beijing China

**Keywords:** Bilirubin, Gut microbiota, Neonatal jaundice

## Abstract

**Importance:**

Understanding the dynamic interplay between gut microbiota development and bilirubin metabolism may provide new insights into the pathophysiology of neonatal jaundice. Identifying microbial taxa associated with bilirubin fluctuations could help inform early prediction and microbiota‐targeted interventions for hyperbilirubinemia.

**Objective:**

To investigate the correlation between dynamic changes in the gut microbiota and bilirubin concentrations during the neonatal period.

**Methods:**

Bilirubin concentrations were monitored daily throughout the neonatal period. Fecal samples were collected from neonates on days 1, 3, 7, 14, 21, and 28 after birth. The composition of the gut microbiome was assessed by 16S rRNA gene amplicon sequencing of the fecal samples. Within‐subject, same‐day associations between transcutaneous bilirubin (TcB) and genus‐level abundance were quantified using a repeated‐measures correlation.

**Results:**

Thirty neonates were included in the final analysis. Among the top‐30 genera, six exhibited false discovery rate significant, same‐day within‐subject associations with TcB under the repeated‐measures correlation framework (|r_rm_| ≥0.30). Changes in the abundances of the genera *Streptococcus* (*r*_rm_ = +0.416, 95% confidence interval [CI] 0.272–0.543, *P* = 2.084 × 10^−7^; *P*‐adj = 3.126 × 10^−6^) and *Rothia* (*r*_rm_ = +0.340, 95% CI 0.187–0.476; *P* = 3.134 × 10^−5^; *P*‐adj = 1.567 × 10^−4^) were positively correlated with bilirubin concentrations throughout the neonatal period. In complementary cross‐sectional analyses centered on meconium, additional genus–bilirubin correlations were identified for TcB measured on postnatal days 3–7 and for the neonatal TcB peak, with multiplicity controlled separately for each endpoint.

**Interpretation:**

A correlation was found between dynamic changes in the gut microbiome and bilirubin concentrations during the neonatal period. The identified genera might be potential markers or targets for intervention for neonatal jaundice.

## Graphical abstract

In the prospective cohort of term neonates, daily bilirubin monitoring combined with serial fecal 16S rRNA sequencing showed that early gut microbiota trajectories are linked to bilirubin dynamics, with distinct bacterial genera associated with higher versus lower peak bilirubin.

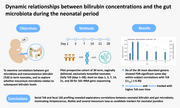

## INTRODUCTION

Neonatal jaundice is a prevalent neonatal condition distinguished by yellow discoloration of the skin and ocular tissues, resulting from elevated bilirubin concentrations, which are a byproduct of normal erythrocyte degradation.^1^ Hyperbilirubinemia (HB) is diagnosed when the serum bilirubin concentrations exceed the 95th percentile for a specific postnatal hour, necessitating immediate clinical intervention. Approximately 10% of term neonates and 25% of preterm neonates meet these criteria for HB annually, which greatly contributes to neonatal hospital admissions and is the primary reason for hospitalization in infants younger than 1 year of age.^2^, ^3^ The severity of this condition is due to its potential to cause irreversible neurological damage, including hearing loss, cerebral palsy, and cognitive impairment, with extreme cases leading to mortality, thereby imposing considerable social and familial burdens. ^4^, ^5^

The intestinal microbiota, which is a complex community of microorganisms, is crucial for the enterohepatic circulation of bilirubin.^6^, ^7^ Research has indicated a major role of the gut microbiota in bilirubin metabolism, with findings from animal models supporting this relationship.^8^, ^9^ Studies using germ‐free rats have shown that the absence of the intestinal microbiota exacerbates hepatobiliary disorders, suggesting that the gut microbiome is a viable therapeutic target for managing bilirubin metabolism disorders.^10^ Short‐chain fatty acids produced by the intestinal microbiota can lower the intestinal pH and inhibit the activity of β‐glucuronidase, thereby preventing the back‐conversion of conjugated bilirubin to its unconjugated form within the gut.^8^

Recent studies have attempted to delineate differences in the composition of the gut microbiota between neonates with jaundice and healthy neonates, although a consensus remains elusive. In 2013, Tuzun et al.^11^ used the polymerase chain reaction technique to assess differences in specific bacterial genera in breast milk and neonatal feces, which were collected between the 14th and 28th days post‐birth. They reported higher concentrations of *Bifidobacteria* in the feces of healthy neonates than in those of jaundice patients. Dong et al.^12^ analyzed meconium in neonates to examine differences in the microbiota between a jaundice group and a control group over the first 42 days. They reported a correlation between a greater abundance of *Bifidobacterium pseudolongum* and a reduced risk of jaundice. In contrast, Duan et al.^13^ observed an increased rate of *Streptococcus* and a reduced rate of *Enterococcus* in neonates with breast milk jaundice. This dynamic interplay between neonatal bilirubin concentrations and the gut microbiota suggests that a more thorough investigation of this relationship will provide insight into potential therapeutic targets.^13^, ^14^, ^15^

This study aimed to examine the dynamic relationship between bilirubin concentrations and the composition of the microbiota to identify changes in the microbiota associated with fluctuations in bilirubin concentrations. We collected fecal samples at various stages of neonatal jaundice development to perform 16S rRNA sequencing.

## METHODS

### Ethical approval

The study followed the ethical guidelines of the 1975 Declaration of Helsinki. Informed consent forms to participate in the study were obtained from the parents or legal guardians. The study protocol was approved by the Ethics Committee of Chinese PLA General Hospital (No. 2012–027).

### Study design

We conducted a pilot, prospective, observational cohort study at the First Medical Center of Chinese PLA General Hospital and the Fourth Medical Center of Chinese PLA General Hospital, Beijing, China. Neonates born between November 2019 and August 2022 were recruited before their mothers’ parturition.

### Participants’ information

All neonates included in the study were from singleton pregnancies and were born via vaginal delivery. The inclusion criteria were as follows: exclusively breastfed, birth weight of 2500–4000 g, gestational age of 37–42 weeks, and Apgar score (at 1, 5, and 10 min) ≥8. The exclusion criteria were as follows: newborns with hemolytic diseases, cephalhematoma, biliary tract obstruction, hereditary metabolic diseases, and infectious diseases. Neonates who received antibiotics, probiotics, prebiotics, or who were formula‐fed were excluded from the final analysis. Furthermore, neonates who provided fewer than two fecal samples during the observation period were also excluded. Clinical data, including gestational age, perinatal complications, sex, birth weight, and Apgar scores, were obtained from the hospitals’ electronic medical records by two independent researchers (Zhongyuan Li and Xi Luo).

### Measurement of bilirubin concentrations

Bilirubin concentrations were estimated using a transcutaneous bilirubinometer (Bilibaby, QBH‐801, Tianjin, China). Transcutaneous bilirubin (TcB) values were assessed simultaneously at the forehead and sternum every 24 hours after birth by a well‐trained guardian. The higher of the two values was used as the result of this measurement. If the TcB value met the diagnostic criteria for neonatal HB,^16^ the neonate was admitted to the hospital for detection of the serum bilirubin concentration and received further treatment.

### Sample collection, DNA extraction, and 16S rRNA sequencing

Meconium (first stool) was collected by trained nurses directly from the diapers into sterile tubes; additional fecal samples were obtained by a trained guardian on postnatal days 3, 7, 14, 21, and 28. Specimens were transported to the laboratory on dry ice and stored at −80°C until processing. DNA extraction, library preparation, and 16S rRNA gene amplicon sequencing were performed at BGI Genomics (Shenzhen, China) under standard procedures. Extraction blanks and PCR negatives were processed in parallel in a sterile hood; no amplification was detected in the controls. The V3–V4 region was amplified with primers 338F (5’‐ACTCCTACGGGAGGCAGCAG‐3’) and 806R (5’‐GGACTACHVGGGTWTCTAAT‐3’), and sequenced on the DNBSEQ G400 platform.

Adapter/primer trimming and paired‐end merging were performed with FLASH^17^ (version 1.2.11), allowing ≤3 mismatches and requiring ≥15‐bp overlap; a 25‐bp sliding window with mean Q20 was applied for quality filtering. Downstream processing used QIIME2^18^ (version 2022.2.0) with q2‐dada2^19^ for denoising, chimera removal, and construction of an exact‐sequence feature table (median 77 626 reads per specimen after QC). Features present in fewer than three samples (≈10% of the cohort) or those with fewer than ten total reads were removed. For alpha‐ and beta‐diversity analyses, samples were rarefied to 20 000 sequences to standardize read depth. Taxonomy was assigned using the q2‐feature‐classifier^20^, ^21^ with a pretrained naïve Bayes model against SILVA^22^ (version 138.1). After standard filtering, 961 of 3442 features remained (173 samples) for subsequent statistical analyses.

### Statistical analysis

Statistical analyses were conducted using the R software (version 4.1.2, https://www.r‐project.org/) and SPSS software (version 26.0; IBM Corp., Armonk, NY, USA). Continuous variables with a normal distribution were expressed as mean ± standard deviation, whereas those with a non‐normal distribution were expressed as median (interquartile range). To evaluate temporal changes in microbial diversity, we analyzed alpha and beta diversities across all samples stratified by sampling time points. Differences in alpha diversity were examined via the Wilcoxon rank‐sum test using the observed features and Shannon index. In addition to significance testing, pairwise effect sizes (Δmean ± 95% confidence interval [CI]) for Shannon diversity were computed using two‐sample *t*‐tests in R (rstatix, version 0.7.2), with Benjamini–Hochberg false discovery rate (FDR) adjustment across comparisons. Differences in beta diversity were examined using the UniFrac distance matrix (ANOSIM, R vegan package, version 2.6‐4). The distance matrix from beta diversity analysis is shown in a principal coordinates analysis (PCoA) plot. The homogeneity of the multivariate dispersions was further assessed with PERMDISP.

Correlation analyses were confined to the 30 genera with the highest mean relative abundance in meconium, thereby fixing a common feature set across time points. For the sample‐by‐genus matrix (indexed by subject and day of life), zeros were replaced by a small pseudocount (1 × 10^−6^), followed by closure to unit sum and a centered log‐ratio transformation to accommodate the compositional nature of the data. TcB measurements were aligned by subject and day and merged with the microbiome matrix. Pairwise complete cases were used, with observations lacking either component excluded.

We defined “dynamic association” as the same‐day (zero‐lag) within‐subject linear synchrony between TcB and the centered log‐ratio (CLR) of genus‐level relative abundance, each expressed relative to the neonate's own mean (person‐mean centering). For each of the top‐30 genera, repeated‐measures correlation (rmcorr) was applied to the mean‐centered TcB and CLR values to estimate a common within‐subject correlation (*r*_rm_). Genera were included only when at least eight subjects and 40 observations contributed. Two‐sided *P*‐values were adjusted for multiple testing across the top‐30 genera using the Benjamini–Hochberg FDR and reported as *P*‐adj. Ninety‐five percent confidence intervals were obtained via Fisher's z‐transformation. Genera with |*r*_rm_| ≥ 0.30 were considered clinically notable. We prespecified two robustness checks: leave‐one‐subject‐out (LOSO) re‐estimation to assess sign stability and effect‐size variability, and within‐subject permutation—shuffling TcB day labels within each neonate—to obtain empirical *P*‐values. The FDR was controlled by Benjamini–Hochberg across the fixed top‐30 genus family.

As a complementary, cross‐sectional summary focused on meconium composition, Spearman's correlations between dominant meconium genera and TcB were computed for two endpoints: (i) TcB measured on each of postnatal days 3–7 (analyzed separately by day). Following a previously published article,^23^ we defined a genus as correlated if the Spearman's correlation coefficients were consistently positive or negative across the five time points and reached statistical significance on at least one time point. (ii) Neonatal peak TcB (maximum across days 1–28). For each endpoint, we reported Spearman's *r* with 95% CI. Multiplicity across genera was controlled separately for each endpoint using the Benjamini–Hochberg procedure, and adjusted *P*‐values (*P*‐adj) were reported. Statistical significance was set at *P*‐adj < 0.05. Given the sample size and design, these analyses were exploratory and hypothesis‐generating, and the results should be interpreted with caution due to limited statistical power and the increased risk of type I error, even after FDR adjustment.

## RESULTS

### Study cohort and baseline characteristics

**FIGURE 1:**
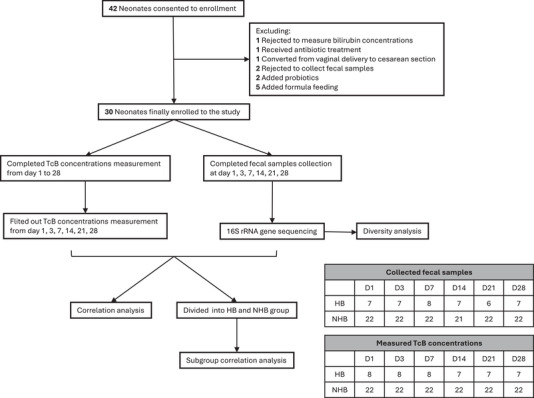
Flowchart of the cohort study. The recruitment process of all the subjects and the subsequent data analysis performed on the collected samples are shown. The quantities of fecal samples and TcB concentration measurements in each group are listed in the table (right) for days 1, 3, 7, 14, 21, and 28. TcB, transcutaneous bilirubin; NHB, non‐hyperbilirubinemia; HB, hyperbilirubinemia; D, day.

Ultimately, 30 neonates were included in this study, and 173 stool samples were collected. The relevant clinical data of the enrolled neonates and mothers are shown in Table 1. Bilirubin concentrations were measured as required in all neonates during the observation period, and the TcB‐day curve (TcB) is shown in Figure S1. All neonates were categorized into the HB group (*n* = 8) or non‐HB group (*n* = 22) according to their TcB values. There were no significant differences in maternal age, days of gestation, parity, maternal height, pre‐pregnancy weight, pre‐pregnancy body mass index, pre‐delivery weight, pre‐delivery body mass index, gestational weight gain, gestational body mass index gain, neonatal sex, or neonatal birth weight between the 2 groups. The study workflow is illustrated in Figure 1.

**TABLE 1:** Baseline characteristics of the study participants

Variables	All (*n* = 30)	HB (*n* = 8)	NHB (*n* = 22)	*P*	*P*‐adj
**Maternal**					
Age (years)	30.50 (2.75)	30.00 (2.00)	31.00 (3.75)	0.619	0.990
Gestation period (days)	276.00 (5.75)	274.50 (4.50)	277.00 (6.75)	0.151	0.694
Parity (primiparae/multiparae)	18/12	5/3	13/9	1.000	1.000
Height (cm)	165.07 ± 4.44	162.62 ± 4.34	165.95 ± 4.23	0.086	0.694
Pre‐pregnancy weight (kg)	56.50 (8.75)	55.50 (6.62)	57.50 (8.75)	0.589	0.990
Weight before delivery (kg)	71.00 (7.00)	71.00 (6.12)	71.00 (7.00)	0.981	1.000
Weight gain during gestation (kg)	14.29 ± 2.87	14.75 ± 3.54	14.13 ± 2.66	0.660	0.990
Pre‐pregnancy BMI (kg/m^2^)	21.50 ± 2.71	21.76 ± 2.86	21.40 ± 2.72	0.765	0.990
BMI before delivery (kg/m^2^)	26.56 (1.97)	26.36 (1.84)	26.62 (2.88)	0.467	0.990
BMI gain during gestation (kg/m^2^)	5.25 ± 1.05	5.56 ± 1.26	5.14 ± 0.98	0.408	0.990
**Neonatal**					
Birth weight (g)	3349.00 ± 384.41	3496.25 ± 317.44	3295.45 ± 399.01	0.174	0.694
Sex (male/female)	13/17	3/5	10/12	1.000	1.000

*Note*: Data are presented as *n*, mean ± standard deviation, or median (interquartile range).

Abbreviations: BMI, body mass index; HB, hyperbilirubinemia; NHB, non‐hyperbilirubinemia; *P*‐adj, *P*‐values adjusted using the Benjamini–Hochberg false discovery rate.

Eight neonates with HB had peak TcB values ranging from 296.1 to 346.8 µmol/L. Total serum bilirubin (TSB) was measured in all eight neonates, and the absolute TSB–TcB differences were 20.6–65.3 µmol/L. For consistency, the TcB values were used for subsequent analyses. In the HB group, one neonate was hospitalized and underwent phototherapy on day 7; stool samples were collected on days 1, 3, and 7 (the day‐7 sample was obtained before phototherapy). The remaining seven neonates did not undergo phototherapy at parental discretion and were managed with home monitoring of TcB together with observation of feeding, alertness, and responsiveness. In all seven cases, TcB declined to the acceptable safety range, and no neurodevelopmental abnormalities or other HB‐related complications were identified during follow‐up.

### Temporal dynamics of the neonatal gut microbiota

**FIGURE 2:**
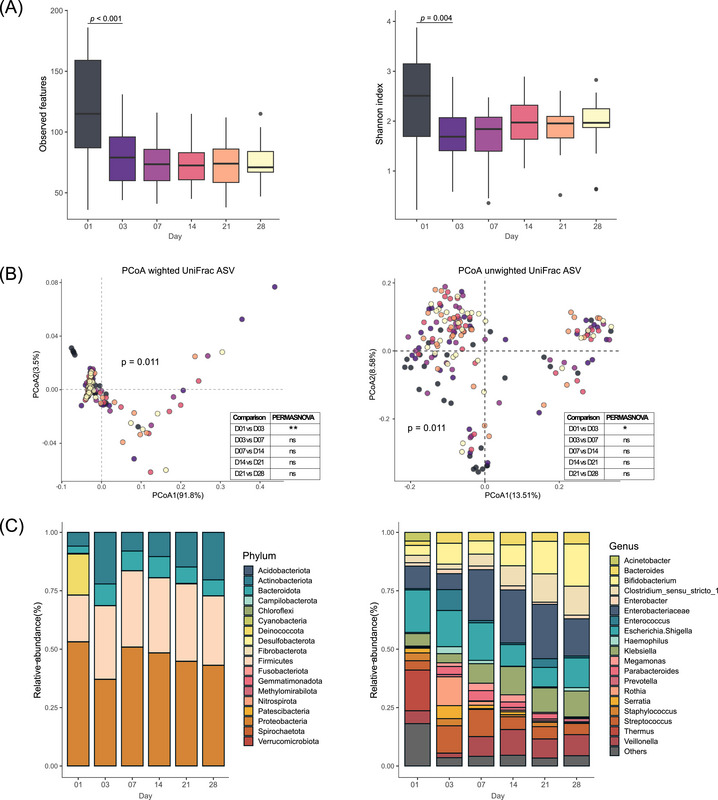
Diversity at the ASV level and composition of the gut microbiota in the neonatal period (days 1–28). (A) Observed features and Shannon diversity of fecal samples collected during the neonatal period. Statistical comparisons between consecutive time points are included. *P*‐values are FDR‐adjusted. Effect sizes (ΔShannon ± 95% confidence intervals) for each pairwise comparison (e.g., D03–D01, D07–D03, etc.) are provided in Table S1. (B) PCoA plots using weighted and unweighted UniFrac distances show microbial community differences at each time point. (C) Composition of the gut microbiome at the phylum and genus levels. ASV, amplicon sequence variant; FDR, false discovery rate; PCoA, principal coordinate analysis.

We performed a longitudinal analysis of the gut microbiota composition across six postnatal time points via 16S rRNA gene sequencing. The meconium microbiome was significantly richer and more diverse than that of the subsequent neonatal stages (Figure 2A, both *P* < 0.005). Specifically, Shannon diversity increased from day 1 to day 3 (Δ = 0.68, 95% CI: 0.23–1.12; *P*‐adj = 0.010), whereas later comparisons showed smaller or non‐significant changes. The detailed effect sizes for all pairwise timepoint comparisons are presented in Table S1. Using unweighted and weighted UniFrac distances, PCoA revealed a distinct composition of the microbiota in meconium, with significant differences in the microbial community of the feces from day 3 onward (Figure 2B, both *P* < 0.05). In addition to UniFrac‐based analyses, we performed an Aitchison distance–based (CLR‐transformed) β‐diversity analysis to account for data compositionality. PERMANOVA showed significant differences in community composition across time points (R^2^ = 0.041, *P* = 0.001; 999 permutations). Group dispersions were heterogeneous (PERMDISP, *P* = 0.001), which was expected during the rapid and variable development of the neonatal gut microbiota. A PCoA plot based on Aitchison distance further illustrated the temporal separation of the samples (Figure S2).

The phyla *Proteobacteria*, *Firmicutes*, *Acidobacteriota*, and *Bacteroidota* dominated the neonatal gut landscape. The relative abundance of the phylum *Deinococcota*, which accounted for a substantial proportion of the meconium sample compared to that at subsequent neonatal time points, indicated a notable change in this microbial population early in the neonatal period. Genus‐level dynamics revealed a progressive increase in the abundance of *Bifidobacterium*, *Clostridium_sensu_stricto_1*, and *Klebsiella* in contrast to a decrease in *Staphylococcus*, *Streptococcus*, and *Escherichia_shigella* over time. These shifts highlight the dynamic nature of neonatal gut colonization (Figure 2C).

### Correlations between gut microbial species and bilirubin concentrations

**FIGURE 3:**
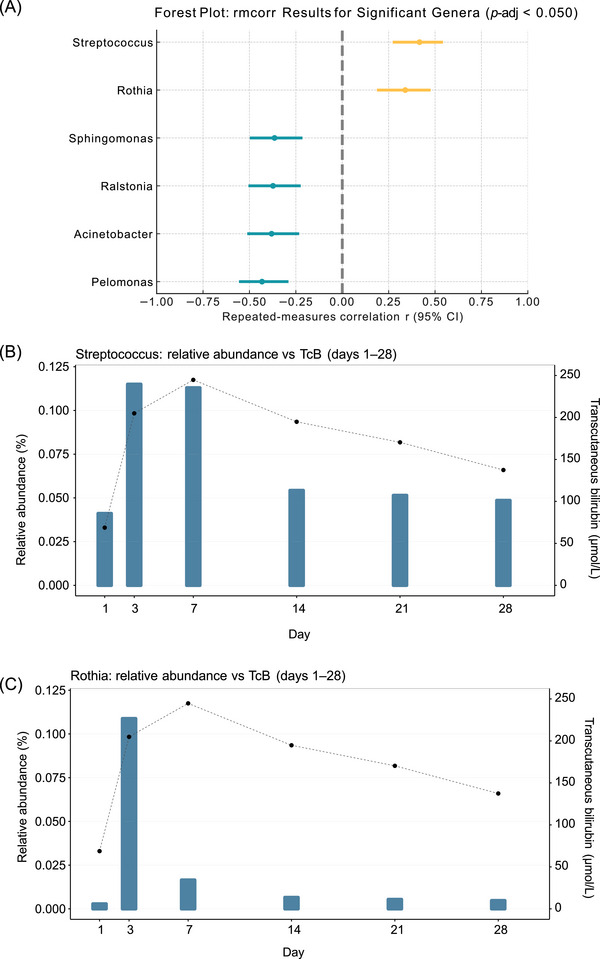
Within‐subject “dynamic” associations between bilirubin and gut genera, and illustrative trajectories. (A) Forest plot showing genera that remained significant after multiple testing in repeated‐measures correlation (rmcorr; |*r*| ≥ 0.30, *P*‐adj < 0.05). Dots represent correlation coefficients (*r*), and horizontal lines indicate 95% confidence intervals (CI); orange and green denote positive and negative associations, respectively. The dashed line marks *r* = 0. Genus‐level relative abundances were closed and CLR‐transformed, and CLR values were paired with the same‐day transcutaneous bilirubin (TcB, µmol/L) within each neonate to estimate zero‐lag within‐subject associations. (B–C) Illustrative time‐series for two positively associated genera—*Streptococcus* and *Rothia*. The x‐axis shows postnatal days 1–28; the left y‐axis indicates mean relative abundance, and the right y‐axis indicates mean TcB. These trajectories are descriptive and not used for inference. rmcorr, repeated‐measures correlation; TcB, transcutaneous bilirubin; CLR, centered log‐ratio; *P*‐adj, *P*‐values adjusted using the Benjamini–Hochberg false discovery rate. *n* = 30 neonates.

To identify fecal genera potentially associated with bilirubin concentrations during the neonatal period, we assessed the correlations between longitudinal changes in the relative abundance of dominant genera and TcB levels over days 1–28. Among the top‐30 genera, six showed FDR‐significant dynamic within‐subject associations with TcB and medium‐sized effects (∣*r*_rm_∣ ≥0.30) (Figure 3A). *Streptococcus*: *r*_rm_ = +0.416, 95% CI 0.272–0.543, *P* = 2.084 × 10^−7^; *P*‐adj = 3.126 × 10^−6^; *Rothia*: *r*_rm_ = +0.340, 95% CI 0.187–0.476; *P* = 3.134 × 10^−5^; *P*‐adj = 1.567 × 10^−4^; *Sphingomonas*: *r*_rm_ = −0.365, 95% CI −0.498 to −0.215; *P* = 6.953 × 10^−6^; *P*‐adj = 4.172 × 10^−5^; *Ralstonia*: *r*_rm_ = −0.373, 95% CI −0.505 to −0.224; *P* = 4.118 × 10^−6^; *P*‐adj = 3.088 × 10^−5^; *Acinetobacter*: *r*_rm_ = −0.381, 95% CI −0.512 to −0.232; *P* = 2.478 × 10^−6^; *P*‐adj = 2.478 × 10^−5^; *Pelomonas*: *r*_rm_ = −0.432, 95% CI −0.556 to −0.290, *P* = 6.260 × 10^−8^, *P*‐adj = 1.878 × 10^−6^. Interpreted under our definition, within the same neonate on the same day, higher‐than‐usual TcB co‐occurred with lower CLR for *Sphingomonas*, *Ralstonia*, *Acinetobacter*, *Pelomonas*, and higher CLR for *Streptococcus*, *Rothia*. Robustness was assessed using two prespecified checks. LOSO re‐estimation preserved the association sign for all six genera with only modest variation in effect size across held‐out neonates (Table S2). Within‐subject permutation (B = 10 000 shuffles of TcB day labels within each neonate) yielded empirical *P*‐values consistent with the primary findings. After Benjamini–Hochberg control across the fixed top‐30 family, both *Streptococcus* and *Rothia* remained FDR‐significant (*P*‐adj < 0.05) (Tables S3 and S4). In line with prior low‐biomass literature, a subset of the associated genera—*Acinetobacter*, *Ralstonia*, *Sphingomonas*, and *Pelomonas—*are frequently flagged as contamination‐sensitive.^24^, ^25^ Accordingly, we present their estimates for completeness but avoid mechanistic interpretation, and instead focus the biological discussion on *Streptococcus* and *Rothia*.

To visualize cohort‐level temporal patterns, we plotted dual‐axis trajectories for *Streptococcus* and *Rothia* across postnatal days 1–28 (Figure 3B–3C). Both genera exhibited cohort‐level profiles that visually tracked the early postnatal rise and subsequent decline of TcB, consistent with the same‐day (zero‐lag) within‐subject positive associations identified by rmcorr. These plots are descriptive and are not used for inference; statistical conclusions rely on rmcorr analyses and robustness checks.

**FIGURE 4:**
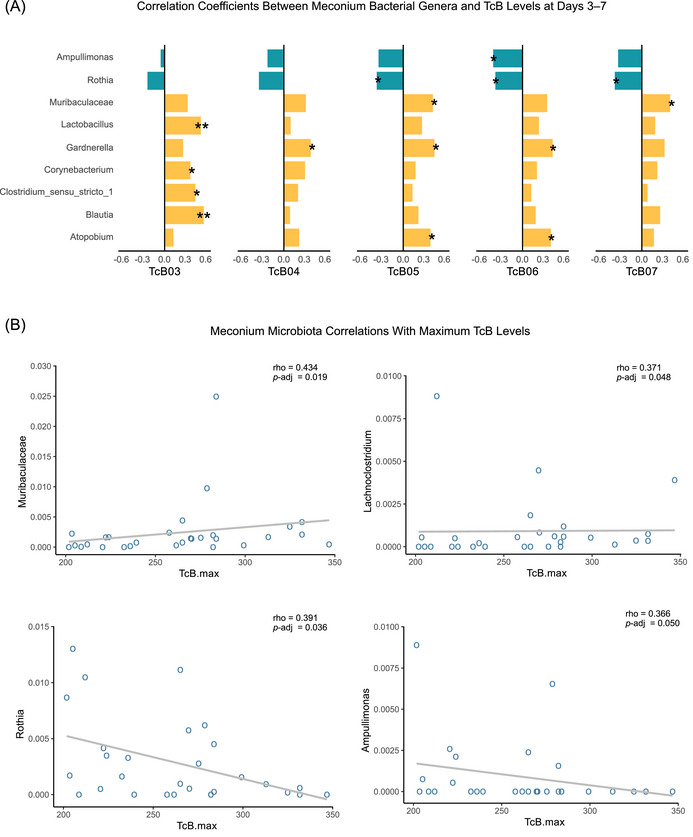
Associations between meconium microbiota and neonatal bilirubin levels. (A) Spearman correlation coefficients between the relative abundance of dominant bacterial genera in meconium and transcutaneous bilirubin (TcB) levels measured on postnatal days 3–7. Bar colors indicate correlation direction (orange, positive; blue, negative). Asterisks denote statistical significance after Benjamini–Hochberg false discovery rate (FDR) correction: *P*‐adj < 0.05 (*), *P*‐adj < 0.01 (**). (B) Scatterplots showing Spearman correlations between the relative abundance of dominant meconium genera and the maximum TcB observed during the neonatal period. Each point represents one neonate (x‐axis, individual maximum TcB; y‐axis, genus‐level relative abundance). Solid lines show fitted linear trends for visualization. Panels report Spearman's *r* and *P*‐adj. TcB, transcutaneous bilirubin. *n* = 30 neonates.

Beyond the early‐life analysis linking bilirubin and the initial stool microbiota, we performed a preliminary exploratory analysis of correlations between dominant meconium taxa and TcB concentrations during postnatal days 3–7 (Figure 4A). This analysis identified positive associations between TcB levels and several genera, including *Muribaculaceae, Lactobacillus, Gardnerella, Corynebacterium, Clostridium_sensu_stricto_1, Blautia*, and *Atopobium*, while *Ampullimonas* and *Rothia* were inversely correlated.

To further assess the clinical relevance of meconium microbiota, we evaluated the association between dominant genera in the meconium and the maximum TcB concentrations recorded during the neonatal period. Significant positive correlations were found for *Muribaculaceae* (*r* = 0.434, 95% CI: −0.066 to 0.605, *P*‐adj = 0.019) and *Lachnoclostridium* (*r* = 0.371, 95% CI: −0.132 to 0.561, *P*‐adj = 0.048), while *Rothia* (*r* = −0.391, 95% CI: −0.509 to 0.203, *P*‐adj = 0.036) and *Ampullimonas* (*r* = −0.366, 95% CI: −0.501 to 0.213, *P*‐adj = 0.050) showed negative correlations (Figure 4B).

## DISCUSSION

In this study, we used a transcutaneous bilirubinometer to capture dynamic changes in bilirubin concentrations throughout the neonatal period. We provided a more continuous and comprehensive dataset compared with previous studies, which typically measured bilirubin levels at only one or two time points. We identified 12 genera that were correlated with the development of neonatal HB through 16S rRNA sequencing. To mitigate potential confounding factors, such as the method of delivery and feeding habits, this study exclusively included full‐term neonates delivered vaginally and exclusively breastfed.

When we extended our analysis to the specific effects of bacterial genera on bilirubin concentrations, we found that the changes in the relative abundances of *Streptococcus* and *Rothia* were positively correlated with changes in neonatal bilirubin concentrations. Following prespecified sensitivity analyses, the within‐subject “dynamic” associations of *Streptococcus* and *Rothia* with TcB remained positive, directionally consistent, and FDR‐significant. These findings were stable to single‐subject leverage and robust to distributional assumptions, indicating that the results are not contingent on model specification or individual outliers. In line with our results, Duan et al.^13^ and Li et al.^15^ reported that neonates with breast milk jaundice exhibited higher relative abundances of *Streptococcus* and *Rothia*, respectively. *Streptococcus* spp. are predominantly homolactic fermenters whose rapid production of lactic acid acidifies the intestinal lumen.^26^, ^27^ In neonates, this acidification is expected to enhance enterohepatic cycling of bilirubin via two coupled effects: first, lowering luminal pH shifts unconjugated bilirubin (UCB) toward its non‐ionized, membrane‐permeable form, facilitating passive reabsorption; second, mildly acidic conditions (≈ pH 5–7) keep many gut‐microbial *β*‐glucuronidases within their functional range, sustaining deconjugation of bilirubin diglucuronide to UCB.^28^, ^29^ Taken together, we conjecture that enrichment of *Streptococcus* and the attendant drop in luminal pH may provide a mechanistic basis for increased bilirubin recirculation in early life. In vitro, *Rothia dentocariosa* activates toll‐like receptor 2 (TLR2) to induce tumor necrosis factor‐alpha (TNF‐*α*).^30^ The ensuing TNF‐*α* disrupts tight junctions and increases intestinal epithelial permeability,^31^ thereby facilitating passive reabsorption of UCB.

Four additional genera also met the rmcorr threshold and remained robust under the leave‐one‐subject‐out and within‐subject permutation analyses. However, *Acinetobacter*, *Ralstonia*, *Sphingomonas*, and *Pelomonas* have been repeatedly annotated as contamination‐sensitive in low‐biomass workflows.^24^, ^25^ We therefore present their estimates for completeness while refraining from mechanistic interpretation. We also avoided post hoc removal and re‐estimation, because changing the genus set would alter the CLR reference and invite composition‐induced shifts and selection bias. Accordingly, our biological interpretation centers on *Streptococcus* and *Rothia*.

The meconium microbiota, as the initial form of the gut microbiota in children, is associated with various diseases in childhood, such as allergies and obesity.^32^, ^33^ Therefore, we further examined the correlation between the microbiota of neonatal meconium and bilirubin concentrations during the neonatal period. We found significant positive correlations between the abundance of *Muribaculaceae*, *Lactobacillus*, and *Blautia* in the meconium and changes in bilirubin concentrations from days 3 to 7 post‐birth. Additionally, similar positive correlations were observed between *Muribaculaceae* and *Lachnoclostridium* and the peak bilirubin concentrations. The beneficial nature of these genera, coupled with their association with higher early bilirubin concentrations, suggests potential, but unidentified, mechanisms affecting bilirubin metabolism. As a dominant genus of intestinal microbiota, *Blautia* has been of particular interest since its establishment for its contribution to alleviating inflammatory diseases and metabolic diseases and for its antibacterial activity against specific microorganisms.^34^ *Muribaculaceae* had a strong capacity to metabolize endogenous and exogenous polysaccharides, could produce short‐chain fatty acids, and had cross‐feeding relationships with *Bifidobacterium* and *Lactobacillus*.^35^ Multiple studies have examined probiotics or prebiotics as adjuncts for preventing and treating neonatal jaundice. The evidence is inconsistent. Several recent studies support a therapeutic role for probiotics in the management of neonatal jaundice: in both term and preterm neonates, probiotic use has been associated with shorter phototherapy duration and reduced length of hospital stay.^36^, ^37^ The probiotic strains and dosing regimens vary considerably across studies. Conversely, other studies reported no significant difference in treatment effect between the intervention and control groups.^38^, ^39^ Meanwhile, trials evaluating probiotics for the prophylaxis of neonatal jaundice have not demonstrated definitive efficacy.^40^, ^41^ A meta‐analysis by Deshmukh et al.,^42^ which included nine randomized controlled trials, revealed that prophylactic probiotic treatment did not significantly reduce the incidence of jaundice. Consequently, the routine use of probiotics for the prevention of neonatal jaundice is not recommended. These findings suggest that the timing of probiotic administration should be carefully considered. Premature intervention with probiotics may not effectively prevent HB in newborns.

Consistent with our meconium‐based observations, You et al.^43^ reported a lower relative abundance of *Rothia* in pathologic jaundice neonates than in controls, supporting a potential protective role for *Rothia* against neonatal jaundice. Mechanistically, that study offered only a brief mucosal‐barrier–oriented explanation for this association. Concordant with this view, an animal study showed that *Rothia* administration promoted repair of damaged intestinal mucosa and reduced epithelial permeability,^44^ thereby constraining the enterohepatic circulation of bilirubin. By contrast, our longitudinal correlation analysis based on stool collected at multiple neonatal time points (Figure 3A,C) revealed a positive association between *Rothia* relative abundance and contemporaneous bilirubin concentrations. This study addresses two distinct questions on different time scales. Our primary estimand is a within‐subject, same‐day (“zero‐lag”) dynamic association between TcB and genus‐level CLR abundance, estimated with rmcorr after closure + CLR processing. This targets synchronous fluctuation around each neonate's own mean and mitigates between‐subject confounding. In parallel, we explored baseline (meconium) associations with subsequent bilirubin windows (days 3–7) and with the maximum bilirubin observed over the neonatal period (TcB_max). These baseline analyses are cross‐subject and predictive in spirit. Because the questions and time windows differ, directional differences across analyses should not be interpreted as contradictions, but as window‐ and estimand‐specific signals. We interpret this pattern as time‐ and context‐dependent rather than uniformly “protective” or “harmful”. Genus‐level 16S profiling masks species/strain heterogeneity that can flip the direction of association, and the compositional nature of relative‐abundance data—together with co‐variation among taxa—can shape marginal correlations, particularly in baseline‐to‐future comparisons. These results should therefore be interpreted cautiously, as the relationship between *Rothia* and bilirubin likely depends on timing, analytical context, and taxonomic resolution.

In this study, we identified a novel correlation between the abundance of *Clostridium_sensu_stricto_1* in neonatal meconium and elevated bilirubin concentrations in newborns, which has not been previously reported in the context of neonatal HB. *Clostridium_sensu_stricto_1* is often associated with colonic inflammation and intestinal barrier dysfunction, and it enhances the expression of colonic *TLR4*, *TLR5*, and *NF‐κB* mRNAs and inhibits the expression of tight junction proteins, such as ZO‐1 and occludin, in the colon.^45^ We hypothesize that *Clostridium_sensu_stricto_1* exacerbates serum bilirubin concentrations by promoting intestinal inflammation and increasing mucosal permeability, thereby facilitating the enterohepatic recirculation of bilirubin. This potential mechanism suggests a complex interplay between the gut microbiota and bilirubin metabolism, highlighting the need for further research into microbial contributions to the pathophysiology of neonatal jaundice.

We detected the presence of *Gardnerella* and *Atopobium*, both of which are common constituents of the female vaginal microbiota, in the neonatal gut. We also identified *Corynebacterium* spp. in the meconium, which likely originated from the mother's urogenital system. These findings suggest that neonates are exposed to maternal vaginal and urogenital microbes during the birth process, leading to colonization in the neonatal gut. This possibility further suggests an effect of the maternal vaginal and urogenital microbiota on neonatal health. Such insights highlight the importance of understanding the transmission and effects of maternal microbes on infants, potentially leading to strategies to manage and optimize microbial effects from birth.^46^, ^47^

While our study raises the possibility of microbiome‐targeted interventions, we did not include an intervention arm. Randomized controlled trials would be required to determine whether altering the gut microbiota (for instance, via probiotic supplementation) can effectively reduce neonatal HB. A recent study in *Microbiome* provides a useful blueprint for subsequent work.^48^ Through stratified and multi‐omics analyses, the study associated early colonization with HB risk, validated *Bifidobacterium* species as bilirubin modulators, and delineated AA/DHA‐mediated mitigation and enrichment mechanisms. Guided by this framework, we will nominate intervention targets among genera correlated with bilirubin levels.

Our study revealed that alpha diversity was greater at the meconium time point than at the later time points, whereas beta diversity exhibited distinct variations. This observation is consistent with previous studies on the initial establishment of the neonatal gut microbiota. Bokulich et al.^49^ reported that phylogenetic diversity in newborns delivered vaginally first decreased but then increased over the first two years. This trend was also noted in exclusively breastfed infants regarding phylogenetic diversity and observed species numbers. In Wang et al.’s study of 28 healthy neonates, the species composition and number of observed features were greater in initial meconium samples than in those taken at 7 and 28 days post‐delivery.^50^ This finding indicated greater species richness in the meconium, as shown by a higher Chao1 index.

Despite the innovative approach of our study, several limitations should be noted. The sample size was small (*n* = 30), which limits statistical power and may increase the risk of type I errors. As such, the results should be interpreted as exploratory and hypothesis‐generating rather than confirmatory. Recruitment was curtailed during the COVID‐19 pandemic, and parental hesitancy further constrained enrollment. To improve internal validity, we applied strict inclusion criteria to minimize confounding from major perinatal factors such as delivery mode and feeding type. This approach increased cohort homogeneity and, in turn, limited generalizability to broader neonatal populations, including preterm and formula‐fed infants. Continuous high‐frequency bilirubin monitoring throughout the first 28 days improved measurement precision and enabled dynamic analyses, but it did not offset the constraints imposed by sample size. Residual confounding from unmeasured factors may remain, including genetic variation in bilirubin‐metabolism genes and environmental exposures such as maternal diet and household microbiota. No neonate in this cohort received postnatal antibiotics, which reduces this potential confounder. Nevertheless, because antibiotics can perturb the neonatal gut microbiome and influence enterohepatic bilirubin cycling, findings may differ in settings with routine antibiotic use. Future studies should explicitly measure and adjust for such exposures.

We also note a statistical limitation in the β‐diversity analyses. PERMDISP indicated significant differences in group dispersions (*P* = 0.001). This result is not unexpected, because variability in microbial composition is naturally greater at some neonatal time points than others. Such dispersion differences may contribute to the PERMANOVA signal, but they likely reflect the biological dynamics of gut microbiota maturation rather than analytical artifacts. Importantly, both UniFrac‐ and Aitchison‐based analyses showed consistent temporal shifts, supporting the robustness of our findings.

Microbiome profiling relied on 16S rRNA gene sequencing without metagenomic or metabolomic assays, which limits taxonomic resolution and constrains mechanistic inference about pathways linking the microbiota and bilirubin. As a result, we could not map compositional changes to functional pathways with high confidence. Given the low biomass of stool at early time points, four genera (*Acinetobacter*, *Ralstonia*, *Sphingomonas*, and *Pelomonas*) were annotated as contamination‐sensitive and reported transparently without mechanistic interpretation. We avoided post hoc removal to preserve a fixed CLR reference and limit selection bias; LOSO and within‐subject permutation support statistical robustness but cannot distinguish contaminant DNA from true signal.

Clinical endpoints were not directly assessed: we did not evaluate whether specific microbiome profiles were associated with the need for phototherapy, signs of bilirubin encephalopathy, or duration of hospital stays. This gap limits clinical interpretation. Future research should enroll larger and more diverse cohorts, implement multi‐omics approaches with standardized measurement of key confounders, and prospectively link microbial features to clinically meaningful outcomes, including prevention of kernicterus, duration of phototherapy, and length of stay. Taken together, these constraints indicate that the present results are preliminary, and they underscore the need for external validation.

In summary, there is a correlation between the gut microbiota and neonatal bilirubin concentrations. *Streptococcus*, *Rothia*, *Muribaculaceae*, *Lactobacillus*, *Blautia*, *Clostridium_sensu_stricto_1*, *Gardnerella*, *Atopobium*, and *Lachnoclostridium* are potential predictive markers or intervention targets for neonatal jaundice. Future studies should validate these findings in larger, more diverse cohorts to ensure that the observed microbiota–bilirubin correlations are generalizable. We are also pursuing functional investigations such as shotgun metagenomic sequencing and metabolomic profiling to identify specific microbial genes and metabolites that mediate the microbiome–bilirubin interaction.

## CONFLICT OF INTEREST

The authors declare no conflict of interest.

## Supporting information



Supporting Information
