# Supplementary material for: Dynamic relationships between bilirubin concentrations and the gut microbiota in the neonatal period: A pilot prospective cohort study
Source: Pediatr Investig. 2025 Dec 5;9(4):347–60. doi: 10.1002/ped4.70032 (PMC12715888; doi:10.1002/ped4.70032)
Supplement: Supplementary file 1 — Supporting Information [file PED4-9-347-s001.pdf]

Supplementary Material for

Dynamic relationships between bilirubin concentrations and the gut microbiota in the neonatal period: A pilot prospective cohort study

Zhongyuan Li, Yan Zhang, Xi Luo, Yangyang Wang, Lihua Peng, Liping Zou

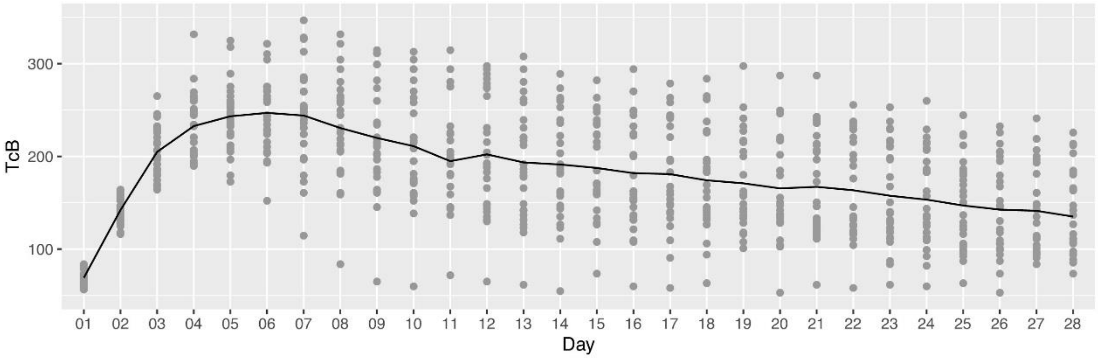

FIGURE S1 Curves of bilirubin concentrations in all neonates during the observation period. TcB, transcutaneous bilirubin.

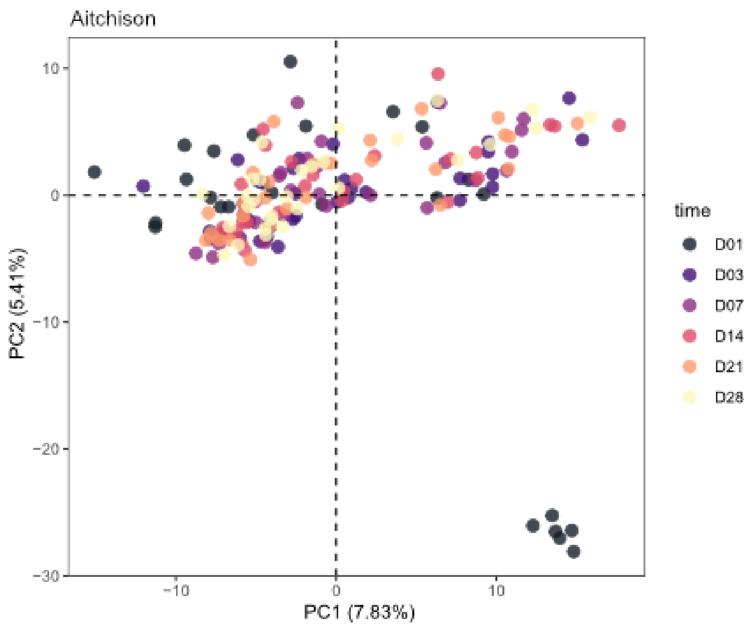

FIGURE S2 PCoA based on Aitchison distances (centered log-ratio transformed abundances). β-diversity differences across time points were tested by PERMANOVA (adonis2, 999 permutations): $R^2 = 0.041$, $F = 1.43$, $P = 0.001$. Group dispersions differed (PERMDISP, $P = 0.001$). $n = 173$ samples from 30 neonates.

TABLE S1. Detailed effect sizes for all pairwise time-point comparisons

Metric	Time-point comparison	Δmean	95% CI	*P*	*P*-adj
Shannon	D03 vs. D01	0.68	0.23 to 1.12	0.004	0.019
Shannon	D07 vs. D03	−0.02	−0.31 to 0.28	0.91	0.91
Shannon	D14 vs. D07	−0.28	−0.56 to 0.00	0.05	0.13
Shannon	D21 vs. D14	0.09	−0.16 to 0.34	0.49	0.76
Shannon	D28 vs. D21	−0.06	−0.30 to 0.18	0.61	0.76
S.obs	D03 vs. D01	35.76	17.79 to 53.73	0.0002	0.0012
S.obs	D07 vs. D03	6.80	−4.11 to 17.71	0.22	0.54
S.obs	D14 vs. D07	0.70	−8.53 to 9.94	0.88	0.96
S.obs	D21 vs. D14	−0.21	−9.78 to 9.35	0.96	0.96
S.obs	D28 vs. D21	−1.74	−10.93 to 7.46	0.71	0.96

Each row represents a genus with corresponding effect size estimates across different sampling time-points. Pairwise effect sizes (Δmean ± 95% confidence intervals [CIs]) for α-diversity metrics (Shannon index and Observed features) across consecutive time points during the neonatal period. Δmean represents the mean difference between time points (later minus earlier). Effect sizes and 95% CIs were obtained from two-sample *t*-tests (rstatix, version 0.7.2), with *P*-values adjusted (*P*-adj) for multiple testing using the Benjamini–Hochberg FDR method.

TABLE S2. Results of leave-one-subject-out (LOSO) re-estimation showing the robustness of associations across neonates.

**genus**	**left_out**	***r***	**same_sign**
Acinetobacter	1	−0.390222604	TRUE
Acinetobacter	2	−0.38838103	TRUE
Acinetobacter	3	−0.369050383	TRUE
Acinetobacter	4	−0.356005123	TRUE
Acinetobacter	5	−0.372104846	TRUE
Acinetobacter	6	−0.384977075	TRUE
Acinetobacter	8	−0.360566486	TRUE
Acinetobacter	9	−0.384142761	TRUE
Acinetobacter	10	−0.369618722	TRUE
Acinetobacter	11	−0.379820654	TRUE
Acinetobacter	12	−0.372094887	TRUE
Acinetobacter	13	−0.409651296	TRUE
Acinetobacter	14	−0.377930468	TRUE
Acinetobacter	15	−0.368570223	TRUE
Acinetobacter	16	−0.407585568	TRUE
Acinetobacter	17	−0.392138411	TRUE
Acinetobacter	18	−0.376675141	TRUE
Acinetobacter	19	−0.378702654	TRUE
Acinetobacter	20	−0.383532196	TRUE
Acinetobacter	21	−0.380880334	TRUE
Acinetobacter	22	−0.402253734	TRUE
Acinetobacter	24	−0.373107083	TRUE
Acinetobacter	25	−0.406695849	TRUE
Acinetobacter	26	−0.379203272	TRUE
Acinetobacter	27	−0.37236802	TRUE
Acinetobacter	28	−0.381133054	TRUE
Acinetobacter	29	−0.388735642	TRUE
Acinetobacter	30	−0.386343774	TRUE
Acinetobacter	31	−0.367622641	TRUE
Acinetobacter	32	−0.360600416	TRUE
Pelomonas	1	−0.419208609	TRUE
Pelomonas	2	−0.439100045	TRUE
Pelomonas	3	−0.452938726	TRUE

Pelomonas	4	−0.409042062	TRUE
Pelomonas	5	−0.424910857	TRUE
Pelomonas	6	−0.437111605	TRUE
Pelomonas	8	−0.429480775	TRUE
Pelomonas	9	−0.433469974	TRUE
Pelomonas	10	−0.421554239	TRUE
Pelomonas	11	−0.408184333	TRUE
Pelomonas	12	−0.446195328	TRUE
Pelomonas	13	−0.445199599	TRUE
Pelomonas	14	−0.428564701	TRUE
Pelomonas	15	−0.444089324	TRUE
Pelomonas	16	−0.437915978	TRUE
Pelomonas	17	−0.43463691	TRUE
Pelomonas	18	−0.427598875	TRUE
Pelomonas	19	−0.425651918	TRUE
Pelomonas	20	−0.464964862	TRUE
Pelomonas	21	−0.440305698	TRUE
Pelomonas	22	−0.417174137	TRUE
Pelomonas	24	−0.436464682	TRUE
Pelomonas	25	−0.435938152	TRUE
Pelomonas	26	−0.407763934	TRUE
Pelomonas	27	−0.427624945	TRUE
Pelomonas	28	−0.450368431	TRUE
Pelomonas	29	−0.435364911	TRUE
Pelomonas	30	−0.410911963	TRUE
Pelomonas	31	−0.457043648	TRUE
Pelomonas	32	−0.416789876	TRUE
Ralstonia	1	−0.360323382	TRUE
Ralstonia	2	−0.380490172	TRUE
Ralstonia	3	−0.362928246	TRUE
Ralstonia	4	−0.345945655	TRUE
Ralstonia	5	−0.364468302	TRUE
Ralstonia	6	−0.405503067	TRUE
Ralstonia	8	−0.397298732	TRUE
Ralstonia	9	−0.376522397	TRUE
Ralstonia	10	−0.394091505	TRUE

Ralstonia	11	−0.352616936	TRUE
Ralstonia	12	−0.36036366	TRUE
Ralstonia	13	−0.393155053	TRUE
Ralstonia	14	−0.394726616	TRUE
Ralstonia	15	−0.381527024	TRUE
Ralstonia	16	−0.380167622	TRUE
Ralstonia	17	−0.364005931	TRUE
Ralstonia	18	−0.385501905	TRUE
Ralstonia	19	−0.363657661	TRUE
Ralstonia	20	−0.374114196	TRUE
Ralstonia	21	−0.373229287	TRUE
Ralstonia	22	−0.371757023	TRUE
Ralstonia	24	−0.375596234	TRUE
Ralstonia	25	−0.364318046	TRUE
Ralstonia	26	−0.349811009	TRUE
Ralstonia	27	−0.381520074	TRUE
Ralstonia	28	−0.365974257	TRUE
Ralstonia	29	−0.378676153	TRUE
Ralstonia	30	−0.353216869	TRUE
Ralstonia	31	−0.366161604	TRUE
Ralstonia	32	−0.365100831	TRUE
Rothia	1	0.330203072	TRUE
Rothia	2	0.326323434	TRUE
Rothia	3	0.326287931	TRUE
Rothia	4	0.318324778	TRUE
Rothia	5	0.344105946	TRUE
Rothia	6	0.344039582	TRUE
Rothia	8	0.306235178	TRUE
Rothia	9	0.324842049	TRUE
Rothia	10	0.335030189	TRUE
Rothia	11	0.336093477	TRUE
Rothia	12	0.337223926	TRUE
Rothia	13	0.386525581	TRUE
Rothia	14	0.372928419	TRUE
Rothia	15	0.343835	TRUE
Rothia	16	0.355700104	TRUE

Rothia	17	0.322791991	TRUE
Rothia	18	0.320677017	TRUE
Rothia	19	0.333840334	TRUE
Rothia	20	0.335488944	TRUE
Rothia	21	0.35934795	TRUE
Rothia	22	0.359871895	TRUE
Rothia	24	0.339379675	TRUE
Rothia	25	0.350344261	TRUE
Rothia	26	0.36502509	TRUE
Rothia	27	0.352901613	TRUE
Rothia	28	0.337846221	TRUE
Rothia	29	0.335053138	TRUE
Rothia	30	0.318247741	TRUE
Rothia	31	0.354006359	TRUE
Rothia	32	0.313547096	TRUE
Sphingomonas	1	−0.349589078	TRUE
Sphingomonas	2	−0.372325697	TRUE
Sphingomonas	3	−0.360489775	TRUE
Sphingomonas	4	−0.336384105	TRUE
Sphingomonas	5	−0.355195335	TRUE
Sphingomonas	6	−0.36855191	TRUE
Sphingomonas	8	−0.38349118	TRUE
Sphingomonas	9	−0.370056151	TRUE
Sphingomonas	10	−0.426428344	TRUE
Sphingomonas	11	−0.363402962	TRUE
Sphingomonas	12	−0.416668116	TRUE
Sphingomonas	13	−0.383911404	TRUE
Sphingomonas	14	−0.4046102	TRUE
Sphingomonas	15	−0.357277745	TRUE
Sphingomonas	16	−0.368211763	TRUE
Sphingomonas	17	−0.35938219	TRUE
Sphingomonas	18	−0.358806561	TRUE
Sphingomonas	19	−0.371570142	TRUE
Sphingomonas	20	−0.369245544	TRUE
Sphingomonas	21	−0.35815545	TRUE
Sphingomonas	22	−0.355420935	TRUE

Sphingomonas	24	−0.355949869	TRUE
Sphingomonas	25	−0.373195878	TRUE
Sphingomonas	26	−0.337130575	TRUE
Sphingomonas	27	−0.350910038	TRUE
Sphingomonas	28	−0.348383589	TRUE
Sphingomonas	29	−0.351442766	TRUE
Sphingomonas	30	−0.34352431	TRUE
Sphingomonas	31	−0.353315736	TRUE
Sphingomonas	32	−0.341844285	TRUE
Streptococcus	1	0.416062158	TRUE
Streptococcus	2	0.414360788	TRUE
Streptococcus	3	0.405097681	TRUE
Streptococcus	4	0.42656725	TRUE
Streptococcus	5	0.427822497	TRUE
Streptococcus	6	0.419150358	TRUE
Streptococcus	8	0.427442964	TRUE
Streptococcus	9	0.426749065	TRUE
Streptococcus	10	0.410564164	TRUE
Streptococcus	11	0.411083624	TRUE
Streptococcus	12	0.425565207	TRUE
Streptococcus	13	0.415943981	TRUE
Streptococcus	14	0.454045649	TRUE
Streptococcus	15	0.41847167	TRUE
Streptococcus	16	0.425728353	TRUE
Streptococcus	17	0.39211293	TRUE
Streptococcus	18	0.434562597	TRUE
Streptococcus	19	0.417785369	TRUE
Streptococcus	20	0.413602519	TRUE
Streptococcus	21	0.415436422	TRUE
Streptococcus	22	0.425323905	TRUE
Streptococcus	24	0.418965465	TRUE
Streptococcus	25	0.408015943	TRUE
Streptococcus	26	0.412391779	TRUE
Streptococcus	27	0.407549595	TRUE
Streptococcus	28	0.41312974	TRUE
Streptococcus	29	0.411423617	TRUE

Streptococcus	30	0.403435988	TRUE
Streptococcus	31	0.380041624	TRUE
Streptococcus	32	0.414024927	TRUE

left_out, ID of the subject left out in this LOSO fold; *r*, repeated-measures correlation (rmcorr) coefficient re-estimated after leaving that subject out; same_sign, whether this fold's *r* has the same sign as the full-data *r* (sign stability).

The table reports effect sizes and their signs for six genera, demonstrating consistency across held-out individuals.

TABLE S3. Empirical *P*-values derived from within-subject permutation tests (10 000 shuffles of transcutaneous bilirubin day labels within each neonate) for the top-30 bacterial families

**genus**	***r***	***P*_emp**	**b**	**B**	***n***	**n_subjects**	***P*-adj**
Acinetobacter	−0.380701874	9.999E-05	0	10 000	173	30	0.00059994
Pelomonas	−0.432188367	9.999E-05	0	10 000	173	30	0.00059994
Ralstonia	−0.372809838	9.999E-05	0	10 000	173	30	0.00059994
Rothia	0.339536327	0.00019998	1	10 000	173	30	0.0009999
Sphingomonas	−0.3647292	9.999E-05	0	10 000	173	30	0.00059994
Streptococcus	0.416445018	9.999E-05	0	10 000	173	30	0.00059994

r, repeated-measures correlation (rmcorr) on centered log-ratio values after within-subject mean centering (full data); *P*_emp, empirical *P*-value from within-subject permutation: (b+1)/(B+1) using two-sided extremity; b, number of permutations with |r_perm| ≥ |r_obs|; B, total permutations (10 000 for all genera); n, total paired observations used (subject × day); n_subjects, number of subjects with ≥2 observations contributing; *P*-adj, Benjamini–Hochberg false discovery rate adjusted *P*-value across the Top-30 genus family, based on *P*_emp.
Values are reported before multiple testing correction.

TABLE S4. Benjamini–Hochberg false discovery rate adjusted empirical *P*-values (*P*-adj) for the within-subject permutation tests.

genus	r_full	LOSO_same_sign_prop	LOSO_*r*_min	LOSO_*r*_max	*P*_emp	*P*-adj	b	B	*r*	n	n_subjects
Acinetobacter	−0.380701874	1	−0.409651296	−0.356005123	9.999E-05	0.00059994	0	10 000	−0.380701874	173	30
Pelomonas	−0.432188367	1	−0.464964862	−0.407763934	9.999E-05	0.00059994	0	10 000	−0.432188367	173	30
Ralstonia	−0.372809838	1	−0.405503067	−0.345945655	9.999E-05	0.00059994	0	10 000	−0.372809838	173	30
Rothia	0.339536327	1	0.306235178	0.386525581	0.00019998	0.0009999	1	10 000	0.339536327	173	30
Sphingomonas	−0.3647292	1	−0.426428344	−0.336384105	9.999E-05	0.00059994	0	10 000	−0.3647292	173	30
Streptococcus	0.416445018	1	0.380041624	0.454045649	9.999E-05	0.00059994	0	10 000	0.416445018	173	30

r_full, full-data repeated-measures correlation coefficient on centered log-ratio with within-subject centering; LOSO_same_sign_prop, proportion of LOSO folds with the same sign as r_full (directional stability); LOSO_*r*_min, minimum *r* across LOSO folds for this genus; LOSO_*r*_max, maximum r across LOSO folds for this genus; *P*-emp, empirical *P*-value from within-subject permutation: (b+1)/(B+1); *P*-adj, BH-FDR *P*-value across the Top-30 genus family based on *P*_emp; b, number of permutations with |r_perm| ≥ |r_obs|; B, total permutations (10 000); *r*, same *r* as in permutation table (for cross-checking); n, total paired observations used (subject × day); n_subjects, number of subjects with ≥2 observations contributing; LOSO, leave-one-subject-out.

Both *Streptococcus* and *Rothia* remained significant after correction (*P*-adj < 0.05).
